# Purification and characterization of novel exopolysaccharides produced from *Lactobacillus paraplantarum* KM1 isolated from human milk and its cytotoxicity

**DOI:** 10.1186/s43141-020-00063-5

**Published:** 2020-10-02

**Authors:** Kanika Sharma, Nivedita Sharma, Shweta Handa, Shruti Pathania

**Affiliations:** Microbiology Research Laboratory, Department of Basic Sciences, Dr Y S Parmar University of Horticulture and Forestry, Nauni, Solan, HP 173230 India

**Keywords:** *L. paraplantarum* KM1, Exopolysaccharides, Purification, Characterization, Cytotoxicity

## Abstract

**Background:**

Microbial origin polysaccharides have gained popularity due to lesser toxicity, better degradability and selectivity as compared to their synthetic counterparts and can be used as emulsifier, stabilizer, thickener, texturizer, flocculating and gelling agent. Here main emphasis on exopolysaccharide production from potential lactic acid bacteria that has GRAS status.

**Results:**

This work was aimed at isolating, purifying and characterizing an extracellular polysaccharide (EPS) produced by a foodgrade lactic acid bacteria *Lactobacillus paraplantarum* KM1. *L. paraplantarum* KM1 was isolated from human milk and identified by conventional and molecular techniques. The 16S rRNA sequence of the isolate was registered in National Centre for Biotechnology Information (NCBI) under accession number KX671558. *L. paraplantarum* KM1 was found to produce EPSs in lactose containing MRS medium, and the maximum yield (47.4 mg/ml) was achieved after 32-h incubation. As evident from TLC and HPLC analyses, the polysaccharide was found to be a heteropolymer-containing glucose, galactose and mannose as main sugars. Different oligosaccharides namely hexoses were obtained after partial hydrolysis of the polymer using MALDI-ToF-MS. The total molecular weight of all polysaccharides present was 348.7 kDa with 100 °C thermal stability as well as water soluble in nature. Cell cytotoxicity revealed that the purified EPS was safe for consumption; thus, it can be used in various food industries as emulsifying and texture agent.

**Conclusions:**

The present study highlighted that exopolysaccharides could be harnessed to improve food products in terms of texture, emulsifying agents, pharmaceutical industry (antioxidants, antitumour, anti-inflammatory and antiviral agents) and as safety purposes.

## Background

Microbial origin polysaccharides based emulsifiers have gained popularity during recent times due to lesser toxicity, better degradability and selectivity as compared to their synthetic counterparts [1]. Apart from this, they have been found to be more compatible with the environment. Exopolysaccharide (EPS) may be produced by micro-organisms as a primary/secondary metabolite during the induced stress conditions of nutrient cycle [2]. Food industry has shown keen interest in generally recognized as safe (GRAS) exopolysaccharides (EPSs) of dairy lactic acid bacterial origin, which are often being used to upgrade the texture and consistency of fermented dairy products [3, 4]. Numerous strains of lactobacillus genera have a potential to produce exopolysaccharide under specific conditions with wide range and diversity of structure and have a potential to be used as nutraceuticals [5, 6]. These macromolecules can be used as stabilizer, emulsifier, thickener, texturizer, flocculating and gelling agent in the wake of their high viscosity and pseudoplastic rheological properties [7–9]. These structurally and functionally diversified biological macromolecules have drawn attention because of their bioactive role and large array of potential applications in pharmaceuticals as antioxidants, antitumour, anti-inflammatory and antiviral agents and as immunomodulant [10, 11].

Investigations on the chemical compositions and molecular structures of EPSs are important to establish their structure–function relationship. Recurring sugar units joined together by glycosidic linkages form EPSs and numerous inter- and intra-molecular hydrogen bonds exists because of hydroxyl groups in the structure [12, 13]. Cross-linking networks of linear or helical chains assemble together to form two- or three-dimensional complex structures for EPSs. These forms are soluble in acidic solutions and neutral aqueous solutions. Researchers still face challenges of explicit characterization of solid- and liquid-state conformations in case of these polymers [14, 15].

To investigate the possible applications of EPS in food and pharmaceutical industry, this research aims to focus on the production, purification and physico-chemical characterization of an EPS produced by *Lactobacillus paraplantarum* KM1 probiotic microorganisms isolated from human milk origin as well as its GRAS status.

## Methods

All of the chemicals used were of analytical reagent grade. Sephadex G75 was obtained from Bangalore Genie Private Ltd (Bangalore, India). The remaining chemicals were procured from Merck Specialities Private Ltd (Mumbai, India).

### Source of culture

KM1 bacterial isolate showing high EPS content was isolated from pooled human milk and was maintained on MRS agar and used throughout the study (Fig. 2a). The culture was maintained by biweekly transfers into sterile litmus at 1% level by inoculated at 37 °C for 24 h and held at − 4 °C between transfers. The sequence analysis of 16S ribosomal RNA gene technique (16S rRNA) was employed for identification of isolate KM1. Then the sequence homologies were analysed by comparative studies using “The National Centre for Biotechnology Information (NCBI)” using web link (http://www.ncbi.nlm.nih.gov/) and Basic Alignment Search Tool (BLAST). The registered accession number of *Lactobacillus paraplantarum* KM1 is KX671558.

### Culture conditions for EPS production

Eighteen-hour-old inoculum (10^8^ CFU/ml) was obtained in MRS medium after inoculating under static condition at 37 °C. EPS production (Fig. 2a) was achieved in optimized MRS medium (pH 6.5) with addition of lactose (1.5%) and ammonium sulphate (2%) at 35 °C for 32 h [1, 11].

### Purification

Centrifugation at 12,000*g* for 20 min at 4 °C was done for 32-h culture, and 0.22-μm membrane filter (Millipore Corp., Cork, Ireland) was used to filter supernatant. Two volumes of 10% (w/v) trichloroacetic acid were added to the filtrate to obtain protein precipitates, and after keeping it overnight at 4 °C under static conditions, it was recentrifuged at 25,000*g* for 20 min. Four volumes of prechilled 95% (v/v) ethanol were added to the supernatant, further keeping it for 24 h at 4 °C and centrifuging at 25,000*g* for 20 min at 4 °C to obtain polysaccharide precipitates. The pellet was further lyophilized to make the EPS dry powder. The EPS powder was dissolved in 5-ml distilled water and dialysed using a 14-kDa cut-off dialysis membrane (Himedia, Mumbai, India). The EPS which was dialysed was relyophilized and assessed for carbohydrate content. Carbohydrate content was spectrophotometrically analysed at 490 nm (750 Lambda Double Beam UV–Vis Spectrophotometer; Perkin Elmer, Shelton, CT, USA) following the phenol–sulphuric acid method [16]. Subsequentially, deproteinized EPS was purified by Sephadex G75 chromatography. Sephadex G75 column (2.5 × 100 cm) was used to mount deproteinized EPS solution (10 mg/ml, 3 ml) as per the reported method [17]. Distilled water elute (3 ml/2 min) was collected automatically, and the carbohydrate content was determined using the phenol-sulphuric acid method with glucose as a standard [16]. The polysaccharide fractions obtained were then pooled, concentrated and lyophilized (Fig. 2b) for further study.

### Characterization

#### Effect of temperature

The effect of temperature change on purified EPS from − 20 to 100 °C was studied after 1 h of incubation, and the carbohydrate content was determined using the phenol-sulphuric acid method with glucose as a standard.

#### Effect of pH

The purified EPS with pH varying from 5.5 to 8 was studied for 1 h of incubation period, and the carbohydrate content was determined using the phenol-sulphuric acid method with glucose as a standard.

#### Stability

The stability study of purified EPS from *L. paraplantarum* KM1 was done at room temperature for time interval of 0, 1, 2, 3, 5, 7, 15 and 30 days, and the carbohydrate content was determined using the phenol-sulphuric acid method with glucose as a standard.

### Antimicrobial test

To study antagonistic potential, food borne/spoilage causing bacteria viz., *Staphylococcus aureus* IGMC, *Listeria monocytogens* MTCC 839, *Leucononstoc mesenteroids* MTCC 107 *Enterococcus faecalis* MTCC 2729, *Clostridium perfringens* MTCC 1739 and *Bacillus cereus* CRI, were used. The test strains were arranged from the Institute of Microbial Technology (IMTECH, Chandigarh, India), Indira Gandhi Medical College (IGMC, Shimla, H.P., India) and Central Research Institute (CRI, Kasauli, H.P., India). All these test strains were revived in nutrient agar medium twice for 24 h at 37 °C before performing experiments, as all these indicators were preserved in 40% glycerol at – 20 °C. Purified EPS from *L. paraplantarum* KM1 showed antimicrobial activity against various test organisms @ 1 O.D culture using agar well diffusion assay. The diameter of zone of inhibition extending laterally around the well was measured, and a clear zone of 1 mm or more was considered positive inhibition.

### Solubility

Solubility of *L. paraplantarum* KM1 EPS in water was determined by following the procedure [18]. A suspension was made by dissolving EPS at rate 50 mg/ml in water with continuous agitation at 35 °C for 24 h. This was followed by centrifugation at 5000×*g* for 15 min, and the collected supernatant (0.2 ml) was precipitated with 3 volumes of ethanol. Again, EPS in the form of precipitate was recovered by centrifugation at 10,000×*g* for 5 min. The resultant material was vacuum dried at 50 °C, and the difference in weight was recorded. The solubility was calculated using the given formula:

$$ \mathrm{Solubility}\ \left(\%\right)=\frac{\mathrm{Total}\ \mathrm{carbohydrate}\ \mathrm{concentration}\ \mathrm{in}\ \mathrm{supernatant}\ }{\mathrm{weight}\ \mathrm{of}\ \mathrm{sample}\ \left(\mathrm{dry}\ \mathrm{weight}\ \mathrm{basis}\right)}\times 100 $$

### Water-holding capacity

Purified EPS was characterized for water-holding capacity (WHC) by suspending 0.2-g sample in 10 ml of deionized water on a vortex mixer [18]. Dispersed material was centrifuged at 10,000 rpm for 25 min. Unbound water that was not held by EPS material was discarded. The entire EPS material was dropped on the pre weight filter paper for complete drainage of water. The weight of EPS thus precipitated was recorded. The percentage of WHC was calculated using the following expression:

$$ \mathrm{WHC}\ \left(\%\right)=\frac{\mathrm{Total}\ \mathrm{sample}\ \mathrm{weight}\ \mathrm{after}\ \mathrm{water}\ \mathrm{absorption}}{\mathrm{weight}\ \mathrm{of}\ \mathrm{sample}\ \left(\mathrm{dry}\ \mathrm{weight}\ \mathrm{basis}\right)}\times 100 $$

### Analysis of EPS hydrolysates

HPLC separation of monosaccharide anthranilic acid derivatives was done to identify their composition as described by Anumula [19] using the services provided by NIPER, Mohali, Punjab, India. Analysis of monosaccharide derivatives was aided by reversed-phase C18 column (ZORBAX 300 SB-C18, 5 ml, 4.6 × 250 mm, Agilent Technologies Pvt. Ltd, Alexandra Point, Singapore) of Agilent 1000 series HPLC (Model no. 1100; Agilent Technologies) equipped with a UV detector. Mobile phase system consisted of a gradient programme of solvent A and solvent B combination following the method described by Anumula [19]. Solvent A contained (v/v) 0.5% phosphoric acid, 0.2% 1-butylamine and 1% tetrahydrofuran mixed in water, while solvent B comprised in (v/v) acetonitrile (50%) and solvent A (50%). The separations were realized using a flow rate of 1 ml/min at 24 °C and 20 μl of each sample was injected at a time.

$$ \mathrm{Concentration}\ \mathrm{of}\ \mathrm{Sugars}\ \left(\frac{\mathrm{mg}}{\mathrm{ml}}\right)=\frac{\mathrm{Area}\ \mathrm{of}\ \mathrm{sample}}{\mathrm{Area}\ \mathrm{of}\ \mathrm{standard}}\times \mathrm{Standard}\ \mathrm{dilution} $$

### MALDI-ToF mass spectrometry

TFA (100 mmol/ml) was used to partially degrade EPS, and aqueous ethanol [80% (v/v)] was helpful in removing acid resistant material from the digest by diluting it. The ethanol soluble oligosaccharides were then lyophilized. The 1-ml deionized water was used to dissolve 1 mg of oligosaccharide. The sample was mixed with equal volume of 2,5-dihydroxybenzoic acid (10 mg/ml) as matrix, prior to MALDI analysis. MALDI analysis was done on an Applied Biosystem Voyager-DE PRO MALDI ToF mass spectrometer with a nitrogen laser (337 nm) operated in an accelerating voltage (20 kV). Every spectrum was obtained in the positive ion reflector mode as an average of 100 laser shots. External calibration of the data was done using angiotensin and ACTH (Applied Biosystem, Carlsbad, CA, USA). The reproducibility of each spectrum was checked five times by comparing it with the prepared sample.

### Surface analysis of purified *L. paraplantarum* KM1 EPS

Surface analysis of purified EPS was studied by scanning electron microscope (SEM) at different magnifications. EPS was fixed on aluminium stub and gold sputtered. The sample was examined under VEGA TESCAN SEM (Brno, Czech Republic) using services provided NIPER, Mohali, Punjab, India.

### Cytotoxicity evaluation

The extracted purified EPS was evaluated for cytotoxicity using mammalian epithelial cell line (HEp-2C) by MTT assay for their safety usage pattern. HEp-2C cells were maintained in Earle’s Minimum Essential Medium (Sigma, USA), supplemented with 10% foetal calf serum (Biosera, UK), 1% non-essential amino acids (Sigma, USA), 2 M l-glutamine (Sigma, USA), 100 μg/ml streptomycin (Sigma, USA) and 100 IU/ml penicillin. By seeding cells in 96-well culture plates (Polylabo, France), MTT assay [20] was repeated three times for each EPS concentration, at the rate of 2.104 cells/well, and treated with increasing concentrations of EPS (0, 100, 200, 300 and 400 μg/ml) for 24 h. The MTT solution (150 μl, 5 mg/ml) was mixed in the culture medium, poured into culture wells, and then, plates were incubated for 3 h at 37 °C. Supernatant was discarded, and the absorbance was measured using an ELISA reader at 540 nm with 690 nm reference. Dose-response curves were computer plotted after converting the mean data values to percentages of the control response. Cultures were then incubated with EPS for 24 h, and the dose of EPS which gave 50% inhibition of live cells (IC50) was derived from the plotted data by linear extrapolation. The data were expressed as mean ± standard deviation (SD) from at least three independent determinations (triplicates) for each experimental point.

### Statistical analysis

Statistical tool applied for the analysis of data was central composite design (CRD).

## Results

### Production and purification of EPS from *L. paraplantarum* KM1

**Fig. 1:**
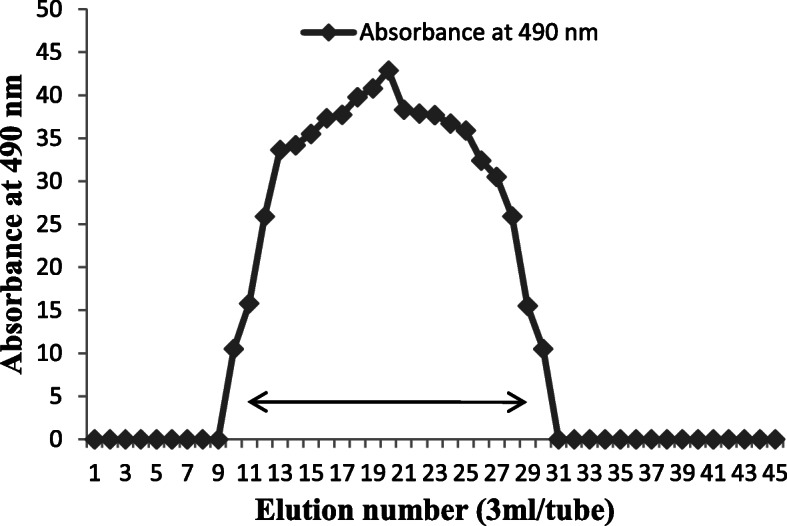
The crude EPS obtained from the treatment with TCA and subsequently with cold ethanol of the growth medium supernatant of *L. paraplantarum* KM1 was fractionated on sephadex G75 chromatography column

**Fig. 2:**
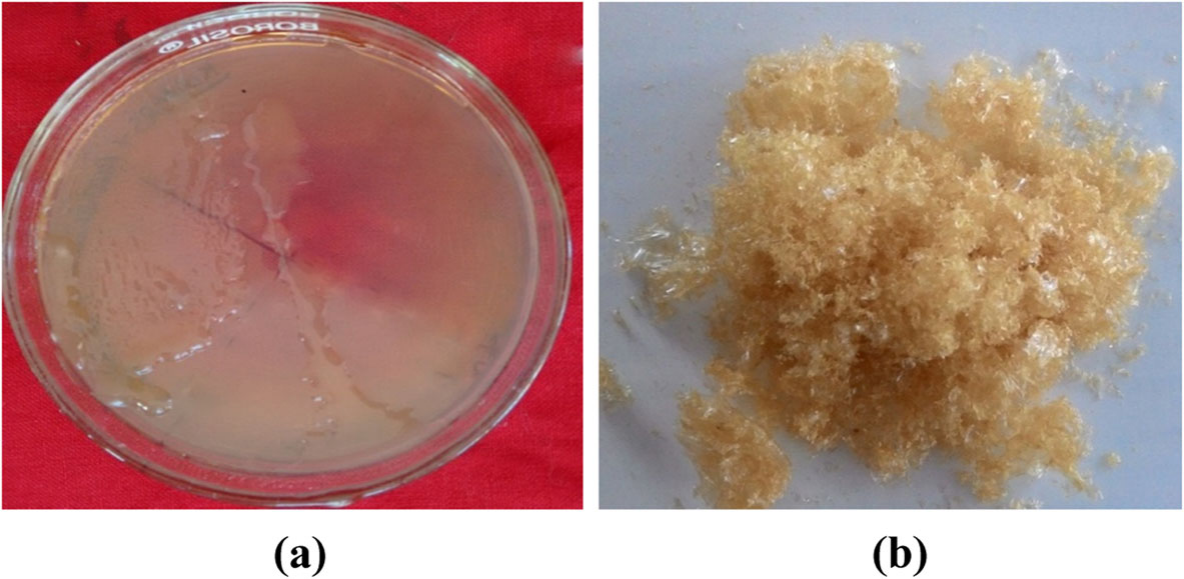
**a** EPS production. **b** The polysaccharide fractions obtained were then pooled, concentrated and lyophilized for further study

The crude EPS obtained from the treatment with TCA and subsequently with cold ethanol of the growth medium supernatant of *L. paraplantarum* KM1 was fractionated on sephadex G75 chromatography column (Fig. 1). The degree of purification and recovery percentage is shown in Table 1. One major peak was eluted with distilled water, and the corresponding fractions containing the neutral polysaccharide were collected for further experiments. The fractions corresponding to the single elution peak of the EPS were collected, concentrated, dialysed and lyophilized to obtain the purified form of EPS, which was used for further physicochemical analysis (Fig. 2).

**Table 1:** Purification profile of exopolysaccharide from *L. paraplantarum* KM1

Purification step	Total volume	EPS concentration (mg/ml)	Total EPS concentration (mg/ml)	%Recovery/yield
**Crude EPS**	30	34.59	1037.7	100
**Dialysis**	25	38.08	952.0	91.7
**Gel exclusion chromatography (Sephadex G-75)**	3	44.01	132.4	12.75

### Characterization of purified EPS

The stability of purified EPS is an intrinsic property dictated by its primary structure; many external factors such as physical factors and chemical reagents influence the overall stability and consequenting activity of EPS. Keeping in view these facts, the effects of various physical and chemical factors viz. pH, temperature, solubility, water-holding capacity, antimicrobial action and stability were evaluated on the behaviour of purified *L. paraplantarum* KM1 EPS.

### Physiological characteristics (independent variables) of purified EPS

**Fig. 3:**
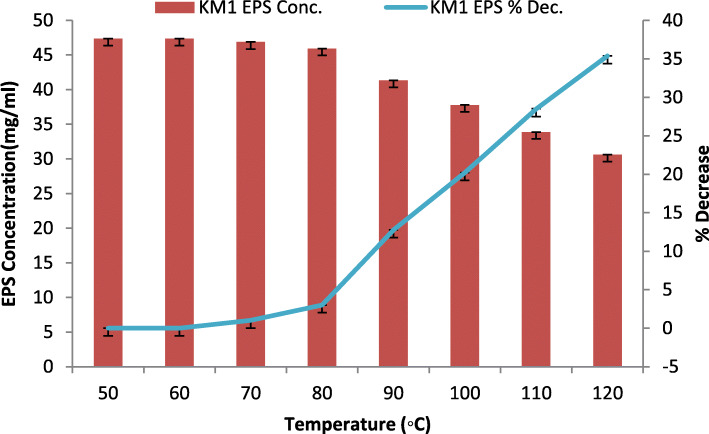
Effect of high temperature resistance on purified EPS concentration

**Fig. 4:**
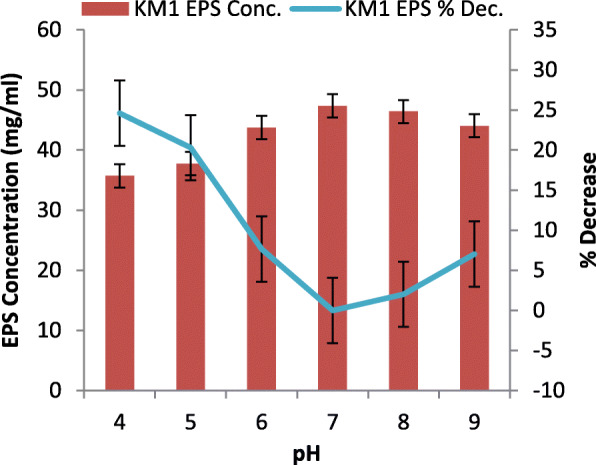
Effect of pH on purified EPS concentration

The effect of high temperature on EPS was studied (Fig. 3). It was noticed that 64.6% of the relative activity (percentage decrease from optimized original activity) was retained by the purified after its treatment at 120 °C temperature while 97.5 % of its relative activity was retained when EPS was treated at − 20 °C of the temperature. The study revealed that low temperature has very little effect on the EPS activity as compared to the high temperature. Similarly, with respect to pH variation, it was observed that EPS retained 75.3% of activity at pH 4.0, whereas 92.9% of its relative activity was retained at pH 9.0 (Fig. 4).

### Storage stability

Shelf stability of the exopolysaccharide was studied at room temperature for 30 days. *L. paraplantarum* KM1 exopolysaccharide was found to be quite stable with marginal decrease 8% after 30 days. Kiran and Chandra [21] studied shelf life of exopolysaccharide from *Bacillus* sp. TSCVKK for 1 month and found it stable at room temperature; however, a loss of 15% exopolysaccharide concentration was observed after 7 days when it was incubated at 30 °C.

### Water-holding activity

The water-holding capacity of purified EPS *L. paraplantarum* KM1 was showed 13.3% (Table 2). It proves that *L. paraplantarum* KM1 EPS are water soluble with good water-holding capacity. These properties are attributed to the permeable structure of polymer chains which can hold large amounts of water through hydrogen bonds [22]. Our results are in accordance using Ahmed et al. [23] who reported good water-holding capacity of its ZW3 EPS.

**Table 2:** Physical properties of *L. paraplantarum* KM1 EPS

Properties	***Percent activity (%)***
**Solubility**	**13.3**
**Water-holding capacity**	**400**

### Antimicrobial activity

The agar well diffusion method was used to study antimicrobial activity of purified EPS of *L. paraplantarum* KM1 against pathogenic microorganisms. In case of *L. paraplantarum* KM1 EPS, *C. perfringens* was found to be the most sensitive to with diameter of zone of inhibition 16.0 mm which was significantly higher (*P* < 0.05) than others, followed by *L. monocytogenes* (14 mm) and *S. aureus* (13 mm) the last one being the most resistant.

### Determination of molecular weight using MALDI-TOF

**Fig. 5:**
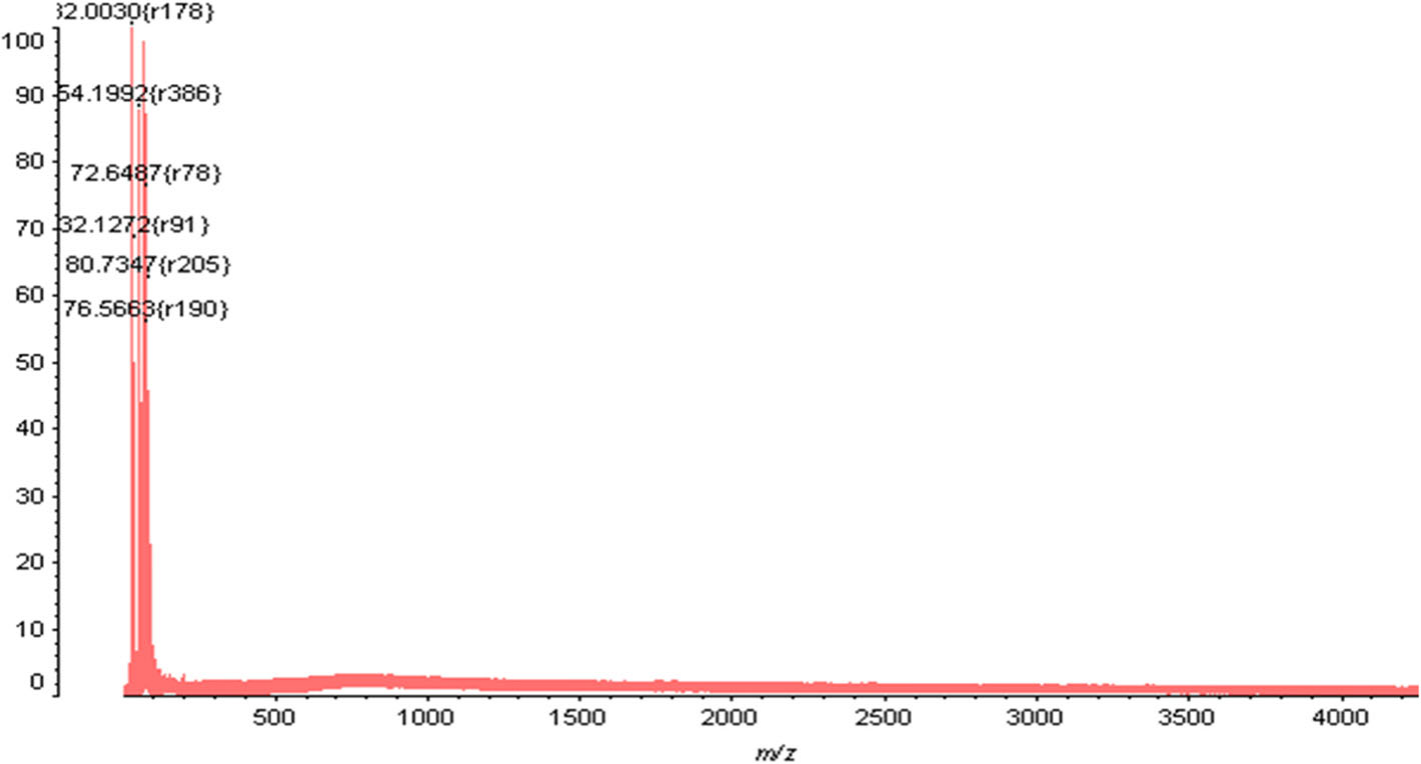
The intense peaks of MALDI-ToF mass spectrum of partially acid-digested EPS

The EPS fractions of the one peak containing high concentration of total sugar were collected for further investigation. The intense peaks of MALDI-ToF mass spectrum of partially acid-digested EPS (Fig. 5) could be assigned to sodiated oligomer peaks. The mass was obtained after MALDI-ToF MS of *L. paraplantarum* KM1 EPS; the masses range found it between 102.67 and 372.97 *m*/*z* which corresponded to the mass of one hexose sugar residue (*m*/*z* 180).

The MALDI-ToF MS of the EPS fraction revealed six peaks with molecular weight of ~32, ~54, ~72, ~32, ~80 and ~76 kDa, respectively with an average molecular weight of 348.7 kDa. The lower molecular weight fractions could mainly be attributed the presence of oligomers present in the polysaccharide.

### Analysis of purified EPS using high-performance liquid chromatography

**Fig. 6:**
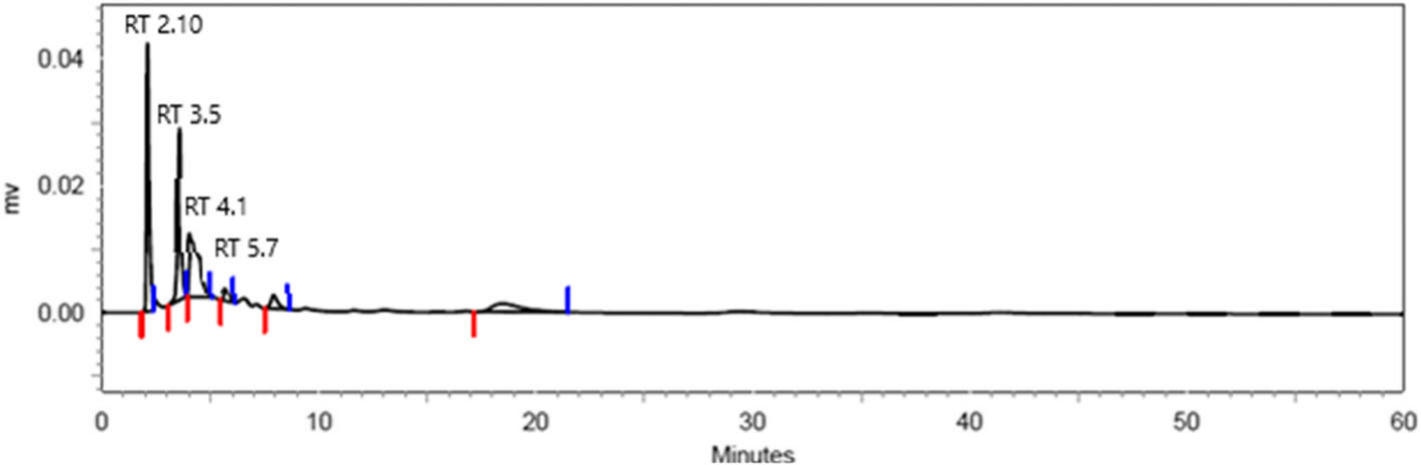
HPLC analysis depicted that EPS contains 615 mg/ml, 22.1 mg/ml and 4.1 mg/ml of glucose, mannose and galactose respectively in purified EPS

Analysis of sugar moieties from hydrolysed EPS was done using thin layer chromatography (TLC). TLC analysis of EPS in *L. paraplantarum* KM1 showed the presence of 3 spots in total. When the computed Rf (Retention factor) value of the spots were compared with the literature Rf value, the compounds were identified as glucose (Rf = 0.18), galactose (Rf = 0.16) and mannose (Rf = 0.21). As TLC results had shown the presence of glucose, galactose and mannose in *L. paraplantarum* KM1, further quantification of these sugars was done by using high-performance liquid chromatography (HPLC). After hydrolysis and anthranilic acid derivatization, EPS I was analysed for its sugar composition by HPLC. The hydrolysed EPS was fractioned by high-performance liquid chromatography (HPLC). The sugar moieties identified were glucose, galactose and mannose with additional sugars which were not traced in TLC analysis. HPLC analysis depicted that EPS contains 615 mg/ml, 22.1 mg/ml and 4.1 mg/ml of glucose, mannose and galactose respectively in purified EPS (Fig. 6 and Table 3).

**Table 3:** Retention factors of hydrolysed EPS of *L. paraplantarum* KM1 from HPLC

Peaks	Retention time	Area	Area percent
1	2.108	364,626	31.67
2	3.583	272,461	23.66
3	4.133	304,724	26.46
4	5.767	32,709	2.84
5	7.925	47,817	4.15
6	18.425	129,122	11.21
Total		1,151,459	100

### Scanning electron microscopic analysis of *L. paraplantarum* KM1 EPS

**Fig. 7:**
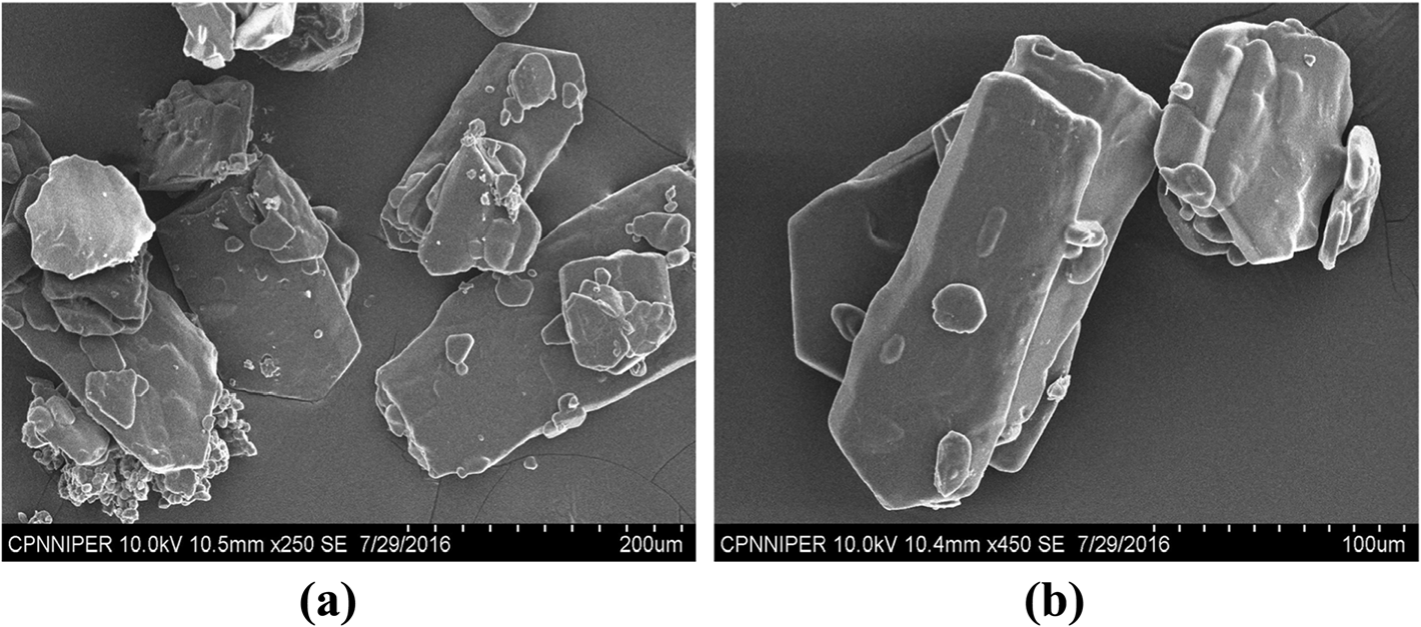
**a** Observation by SEM of the EPS from *L. paraplantarum* KM1. **b** At higher magnification, additional details of microstructure of EPS were visible

Observation by SEM of the EPS from *L. paraplantarum* KM1 revealed a compact structure composed of similar sizes of cubes (Fig. 7a). At higher magnification, additional details of microstructure of EPS were visible (Fig. 7b).

### Cytotoxicity test

**Fig. 8:**
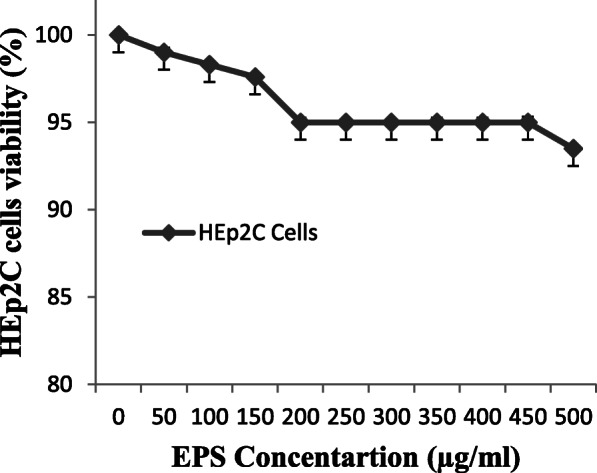
HEp-2C was expressed by the percentage of cell viability after 24h exposure to each purified EPS concentration of *L. paraplantarum* KM1

The *in vitro* cytotoxicity against mammalian epithelial cell line viz. HEp-2C was expressed by the percentage of cell viability after 24-h exposure to each purified EPS concentration of *L. paraplantarum* KM1 (Fig. 8). Only small differences between the negative control (0 μg/ ml EPS) and samples were detected in the cell viability of the cells lines. Even when exposed to 500 μg/ml of EPS, the viability of HEp-2C cells remained unaffected, which was significantly tested (*p* < 0.05).

## Discussion

KM1 EPS is water soluble with good water-holding capacity. These properties are attributed to the permeable structure of polymer chains which can hold large amounts of water through hydrogen bonds [22]. Our results are in accordance with Ahmed et al. [23] who reported good water-holding capacity of its ZW3 EPS produced by *Lactobacillus kefiranofaciens* ZW3 isolated from Tibet kefir. Due to good holding capacity of KM1 EPS, it can be used in various fermented food products for their texture enhancement.

Antimicrobial activity was determined by measuring the diameter of zone of inhibition, indicating that the purified EPS was inhibitory against the test organisms. The zones of inhibition of indicator organisms tested ranged from 13 to 16 mm in diameter. The antimicrobial activity of purified EPS was thought to be a significant incident for competitively excluding or inhibiting the activities of pathogenic intestinal microflora making the host safer against the harmful microorganisms [24].

MALDI-TOF analysis of the EPS fraction was completed. The resultant mass spectrum contained an *x* axis representing *m*/*z* (mass divided by charge) and a *y* axis representing absolute intensity. Similar results of EPS were confirmed by Chowdhury et al. [2] using haxose mass of 180 *m/z* in purified EPS from *Bacillus megaterium* RB-05. Similarly, Liang et al. [25] studied the molecular weight of the purified EPS from *Paenibacillus mucilaginosus* TKU032 using MALDI-ToF MS and revealed two mass peaks with molecular weight of 105 and 135 kDa. The difference in molecular weights of the EPSs could be attributed to the different producing strains, the culture media and the growth conditions used [26]. The molecular weight of an exopolysaccharide plays a role in immune function and performance of the food harbouring the polymer; the present characterization study becomes extremely important [27].

In TLC, the retention times (RT) of monosaccharide compositions of EPS were determined comparing with the chromatograms of each respective standard monosaccharide. The presence of sugars in EPS has been confirmed in several studies. The EPS from *L. plantarum* C88 was composed of galactose and glucose [28]. *L. plantarum* KF5 produced two EPS, both containing glucose mannose and galactose in varied ratios [29]. In our study, the purified EPS of *L. paraplantarum* KM1 was found to contain high concentration of glucose in its EPS.

SEM is considered to be a powerful tool to study the morphological features of polysaccharides and could be used to elucidate their physical properties [6]. The EPS from *L. paraplantarum* KM1 had a smooth cube surface, and additionally, the SEM scan showed that the EPS was made of homogeneous matrix. Similar structure was observed where the structure of their EPS was smooth and cubic [30] and was different from KF5 EPS where the structure was porous [31]. Since many bioactive molecules used in food (i*.*e*.* butylated hydroxyanisole and butylated hydroxytoluene) have been demonstrated to be cytotoxic [32], the current attention is on non-toxic food components. According to the present results, purified EPS concentration of *L. paraplantarum* KM1 was free from the cytotoxic effects and could be of importance in food industry. Similarly, Challouf et al. [33] also studied the effect of purified EPS from *Arthrospira platensis* on colon cancer cell lines ‘Caco-2’ and kidney cell lines ‘Vero’ and reported that their EPS have no cytotoxic effect. El-Deeb et al. [34] performed that in 48 h of treatment, cellular viability inhibition percentage (neutral red quantification results) of MCF7 and CaCo-2-treated cells reached 71.86 and 80.65, respectively with 78.95 and 87.27106 percentage of inhibition, in cellular proliferation (BRDU cellular incorporation); significantly different from the non-treated cells (p < 0.05).

## Conclusions

*L. paraplantarum* KM1 was isolated and identified from human milk. *L. paraplantarum* KM1 was found to produce an efficient amount of EPS and was purified using gel chromatography. Acidic hydrolysis, HPLC and MALDI-TOF MS studied reviled glucose, galactose and mannose sugar moieties in purified EPS. Microstructural characterization of the purified EPS indicated that the EPS had a highly compact structure with smooth cube surface, facilitating formation of films. Future studies will be focused on further characterization of the EPS structure, its biological functions and possible roles in determining the potential probiotic properties of the strain in question.

## Data Availability

All data generated or analysed during this study are included in this article

## Abbreviations

EPS: Exopolysaccharides
h: Hours
°C: Degree Celsius
mm: Millimetre
mg: Milligramme
ml: Millilitre
Kv: Killovolt
nm: Nanometre
μl: Microlitre
